# Shorter sleep durations in adolescents reduce power density in a wide range of waking electroencephalogram frequencies

**DOI:** 10.1371/journal.pone.0210649

**Published:** 2019-01-22

**Authors:** Irwin Feinberg, Ian G. Campbell

**Affiliations:** University of California, Davis, Department of Psychiatry and Behavioral Sciences, Davis, California, United States of America; Imperial College London, UNITED KINGDOM

## Abstract

Despite sleep’s recognized biological importance, it has been remarkably difficult to demonstrate changes in brain physiology with reduced sleep durations. In a study of adolescents, we varied sleep durations by restricting time in bed for four nights of either 10, 8.5 or 7 h. Shorter sleep durations significantly decreased waking electroencephalogram (EEG) power in a wide range of frequencies with both eyes closed and eyes open in central and occipital leads. These findings suggest new research directions and raise the possibility that waking EEG power density could provide a non-invasive test for biologically sufficient sleep.

## Introduction

One long-standing and intuitively plausible hypothesis regarding the biological function of sleep is that it is a period of reduced neural-metabolic activity that permits restoration of substrates required for waking brain functions [1]. Although rapid eye movement (REM) sleep is characterized by normal or even elevated neural activity [2], direct measurements of glucose uptake in humans show that in NREM sleep brain metabolism declines below waking levels by more than 30% [3, 4]. Since NREM makes up 75–80% of human sleep after infancy, this reduced metabolic activity is consistent with the hypothesis that sleep provides or permits replenishment of energy substrates needed for waking neuronal activity. One might therefore expect enforced sleep reductions to impair brain energetics during subsequent waking but such effects have not clearly been demonstrated in human subjects. We report here that graded reductions of sleep durations in adolescents reduce EEG power during waking. These findings were obtained in the course of longitudinal experiments designed to measure sleep need in adolescents.

## Materials and methods

The University of California Davis IRB approved this study, approval number 359498. Participants’ parents provided written consent, and participants older than 12 years provided written assent.

In our 3-year longitudinal study of sleep need in adolescence, we modified sleep durations with 3 different time-in-bed (TIB) schedules consisting of 10, 8.5 and 7 h in bed, each maintained for four consecutive nights. Annually, each S completed each of these 4-night TIB protocols. On the day following the fourth night of each prescribed sleep schedule, participants spent a weekend day in the laboratory for sleepiness and performance tests. The test battery included waking EEG recordings during the Alpha Attenuation Test (AAT—[5, 6]) which gave rise to the observations we report here. Data from this study are archived at the Harvard Dataverse, https://doi.org/10.7910/DVN/EVNUZS.

### Study participants

As this information has been published in detail [7], it will only be briefly summarized. Subjects were recruited who met the initial age (10–14 years), residence (within 20 miles of the sleep lab), medical and sleep health criteria. Parents provided informed consent, and children older than 12 years provided assent. The 77 participants who completed year 1 of the study consisted of 41 boys and 36 girls and had a mean (+/- SD) age of 12.2 +/- 1.2 years at their first recording. Attrition reduced this cohort to 76 participants in year 2 and to 67 in year 3.

### Sleep EEG recording and analysis

On the second and fourth night of the prescribed 4 night TIB schedules, all-night EEG was recorded in the participants’ homes, i.e. in their typical sleep environments. EEG was recorded from electrodes at F3, F4, C3, C4, P3, P4, O1 and O2 with mastoid electrodes, A1 and A2. Electrooculogram was recorded from LOC and ROC referred to a forehead electrode. Bipolar submental electromyogram was also recorded. Signals were recorded versus a reference electrode and electrode pairs such as C3/A2 were obtained by subtraction. Signals were amplified and digitized (400 Hz) with Grass Aura ambulatory recorders. Filter details have been previously published [8].

### Waking EEG recording and analysis

On the morning following the fourth experimental night of each schedule, participants reported to the sleep laboratory at 0830 for a battery of sleepiness tests. These included the multiple sleep latency test and the psychomotor vigilance test, the Alpha Attenuation Test, questionnaires and other measures. The AAT was performed every 2 hours with approximately 1 hour breaks, producing 4 tests per day: 0900, 1100, 1300, and 1500. In the AAT, waking EEG was recorded for 3 min with eyes open while participants stared at a dot on the wall. They then closed their eyes for 2 minutes, opened their eyes and stared at the dot for another 2 minutes, and finally closed their eyes for an additional 2 minutes. Data from O1 and C3 are the focus of this report; virtually identical data obtained from O2 and C4 are reported in the supplemental information. When performing the AAT, participants pressed a button that added an event mark to the EEG recording at each eyes open/closed change. Participants were also monitored with video cameras to determine compliance with the eyes open/closed instructions.

All-night EEG from the second and fourth experimental nights was scored for sleep stages as previously described [7]. Waking EEG recordings from the AAT were analyzed in 5 second epochs using PASS Plus (Delta Software, St. Louis) fast Fourier transform (FFT). FFT parameters were as follows: 2.56 Welch tapered windows with a 1.31 second overlap, yielding 4 windows per 5 second epoch. FFT resolution was 0.391 Hz. A computer program automatically detected low frequency movement artifacts and high frequency EMG or electrical noise artifacts. Epochs containing such artifacts were not included in analysis. Power was averaged separately for eyes open and eyes closed for all artifact-free epochs in each of the 4 recordings across the day. AAT results were not included in the data pool if they contributed fewer than six artifact-free epochs. Bins were summed into the following frequency bands: delta (0.98–4.10 Hz), theta (4.10–8.01 Hz), alpha (8.01–11.91 Hz), beta 1 (11.91–16.99 Hz), and beta 2 (16.99–29.88 Hz). We use the traditional Greek letters for the main EEG frequency bands.

### Statistical analysis

EEG FFT power data were log transformed prior to mixed effects analysis [9]. Following an initial analysis that found significant (p<0.0001) interactions between frequency band and other factors, the 5 main waking EEG frequency bands were analyzed separately. Also, C3, C4, O1, and O2 data were analyzed separately. Linear mixed effect analysis was used to test for effects of TIB, age, and eyes (with interactions) with time of day effects accounted for. In these analyses TIB was treated as a fixed and random variable; age, eyes, and time of day were fixed variables. TIB effects on night 4 sleep stage durations were evaluated with mixed effects analysis with TIB as a fixed and random factor and age as a fixed factor.

Graphs show EEG power density rather than the log power values that were used for statistical analysis. For graphing, data were initially averaged for each subject across the 4 daily trials and across the 3 years of the study. The data points on the graphs are averages of the 77 subjects +/- standard error.

## Results

The continuous sleep EEG recordings on the second and fourth nights of each TIB condition documented that controlling Table 1 shows that varying TIB produced the expected effects on sleep durations. TIB reduction significantly reduced total sleep duration (F_1,76_ = 2024, p<0.0001), REM sleep duration (F_1,76_ = 376, p<0.0001), and NREM sleep duration (F_1,76_ = 1042, p<0.0001). TIB reduction significantly reduced N2 sleep duration (F_1,76_ = 1011, p<0.0001), but not N3 sleep duration (F_1,76_ = 3.28, p = 0.074) which showed a trend toward increasing with decreased TIB.

**Table 1:** Sleep durations. Mean (± se) sleep duration (minutes) for the 3 TIB conditions.

	TIB 7h	TIB 8.5h	TIB 10h
Total Sleep Time	405 ± 2	471 ± 2	531 ± 3
REM Sleep	88 ± 2	111 ± 2	129 ± 2
NREM Sleep	316 ± 2	360 ± 2	403 ± 3
N2 Sleep	203 ± 2	250 ± 3	292 ± 3
N3 Sleep	114 ± 2	110 ± 3	111 ± 2

**Fig 1:**
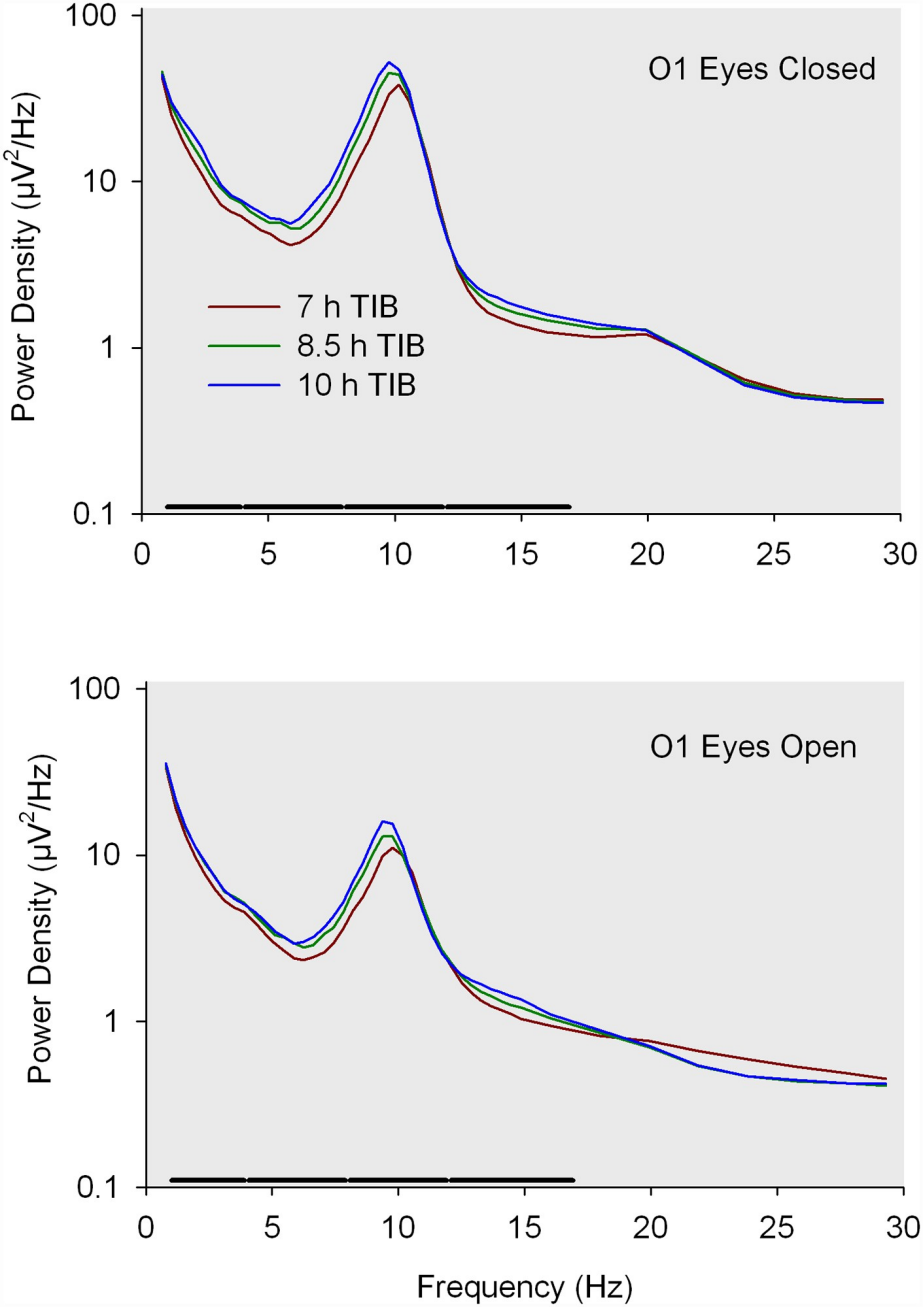
O1 power spectra. Waking EEG power spectra for O1 with eyes closed and eyes open on the day following 4 consecutive nights of 3 different TIB schedules. Thick bars above the x-axis indicate a significant (p<0.0001) TIB effect. For example, TIB significantly affected eyes closed EEG power in the 12–17 Hz band but not the 17–30 Hz band.

An initial analysis of the waking EEG data tested for overall effects across all 5 frequency bands. It showed that longer TIB was associated with higher power density (F_1,76_ = 83.4, p<0.0001) and that the TIB effect differed by frequency band (F_44,2.2x10_^5^ = 33.3, p<0.0001). As shown in Fig 1, EEG power density (PD) in most frequencies in O1 was highest after 10 h in bed and lowest after 7 hours, with the 8.5 TIB condition intermediate. PD was greater with eyes closed than with eyes open in central as well as occipital leads.

**Fig 2:**
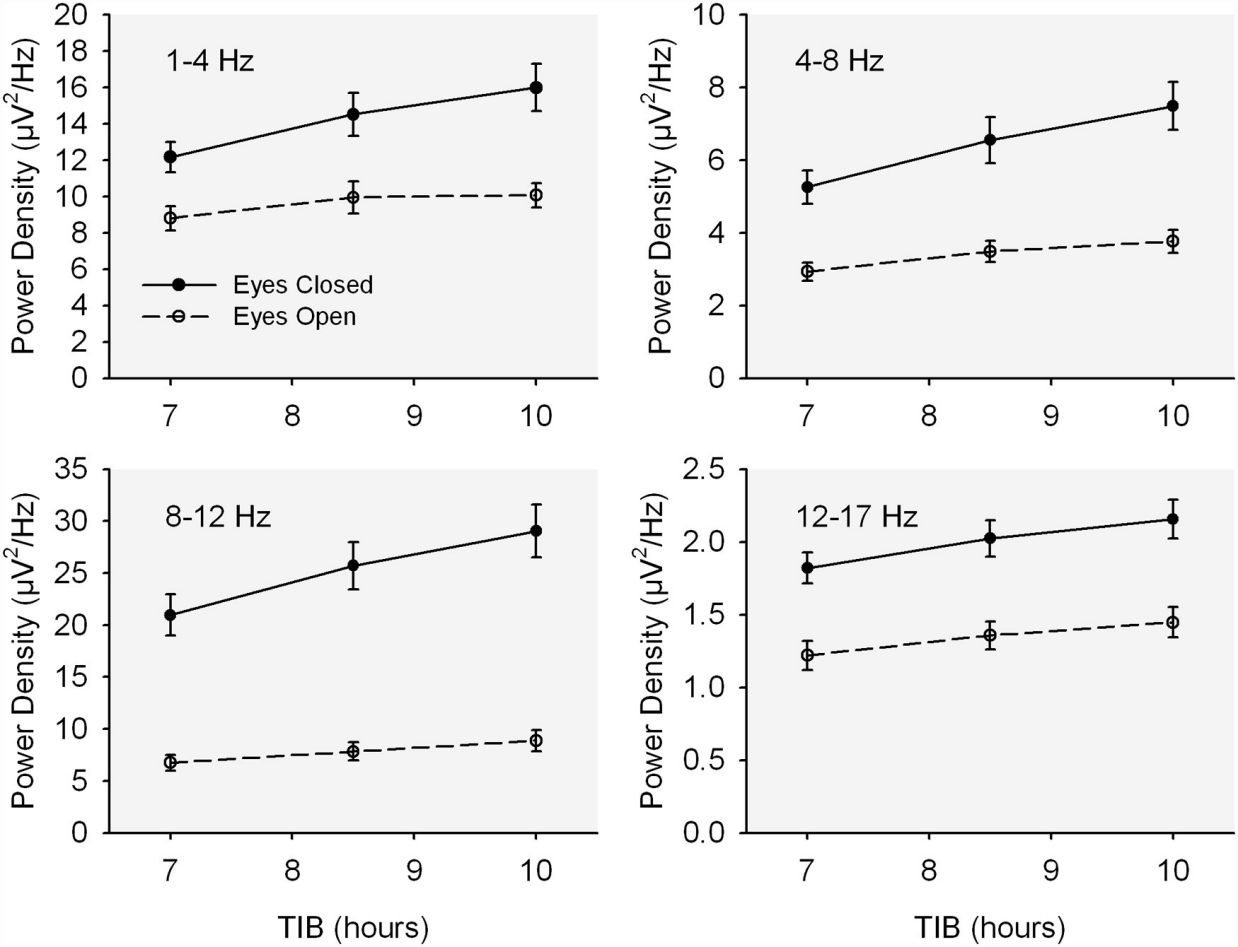
O1 power vs. TIB. The effect of time in bed (TIB) on mean (+/- se) O1 waking EEG power density in four frequency bands for both the eyes closed (solid line, filled circles) and eyes open (dashed line, open circles) conditions.

Table 2 summarizes the tests of statistical significance for O1 and for C3 EEG. (As mentioned above, results for right-sided scalp leads (O2 and C4) were virtually identical to those of the left side and are presented in the Supplement). In O1, log EEG power was significantly reduced by TIB restriction for all frequency bands from delta through beta 1 with both eyes open and eyes closed (Fig 2); also, log power was significantly higher with eyes closed. Beta 2 log power in O1 was not significantly affected by sleep restriction although it did show a strongly significant increase with eye closure. As indicated by the significant TIB by eyes interaction and as shown in Fig 2, the TIB effect on delta, theta, and alpha EEG log power was stronger in the eyes closed condition.

**Table 2:** O1 and C3 statistical analysis. Mixed effects analysis of time in bed (TIB), age, and eyes closed effects on log power of waking EEG recorded from O1 and C3. Significance level is bold for positive effects (e.g. increasing power with increasing TIB), italicized for negative effects (e.g. decreasing power with age), and plain text for non-significant (α = 0.01) effects.

O1				
Band	TIB	Age	Eyes closed	TIB X eyes
Delta 1–4 Hz	**<0.0001**	*<0*.*0001*	**<0.0001**	**<0.0001**
Theta 4–8 Hz	**<0.0001**	*<0*.*0001*	**<0.0001**	**0.0010**
Alpha 8–12 Hz	**<0.0001**	*<0*.*0001*	**<0.0001**	**<0.0001**
Beta 12–17 Hz	**<0.0001**	*<0*.*0001*	**<0.0001**	0.35
Beta 17–30 Hz	0.031	*<0*.*0001*	**<0.0001**	0.35
C3				
Band	TIB	Age	Eyes closed	TIB X Eyes
Delta 1–4 Hz	0.022	*<0*.*0001*	**<0.0001**	0.054
Theta 4–8 Hz	**<0.0001**	*<0*.*0001*	**<0.0001**	0.052
Alpha 8–12 Hz	**<0.0001**	*<0*.*0001*	**<0.0001**	**<0.0001**
Beta 12–17 Hz	**<0.0001**	*<0*.*0001*	**<0.0001**	0.25
Beta 17–30 Hz	0.013	*<0*.*0001*	**<0.0001**	0.23

**Fig 3:**
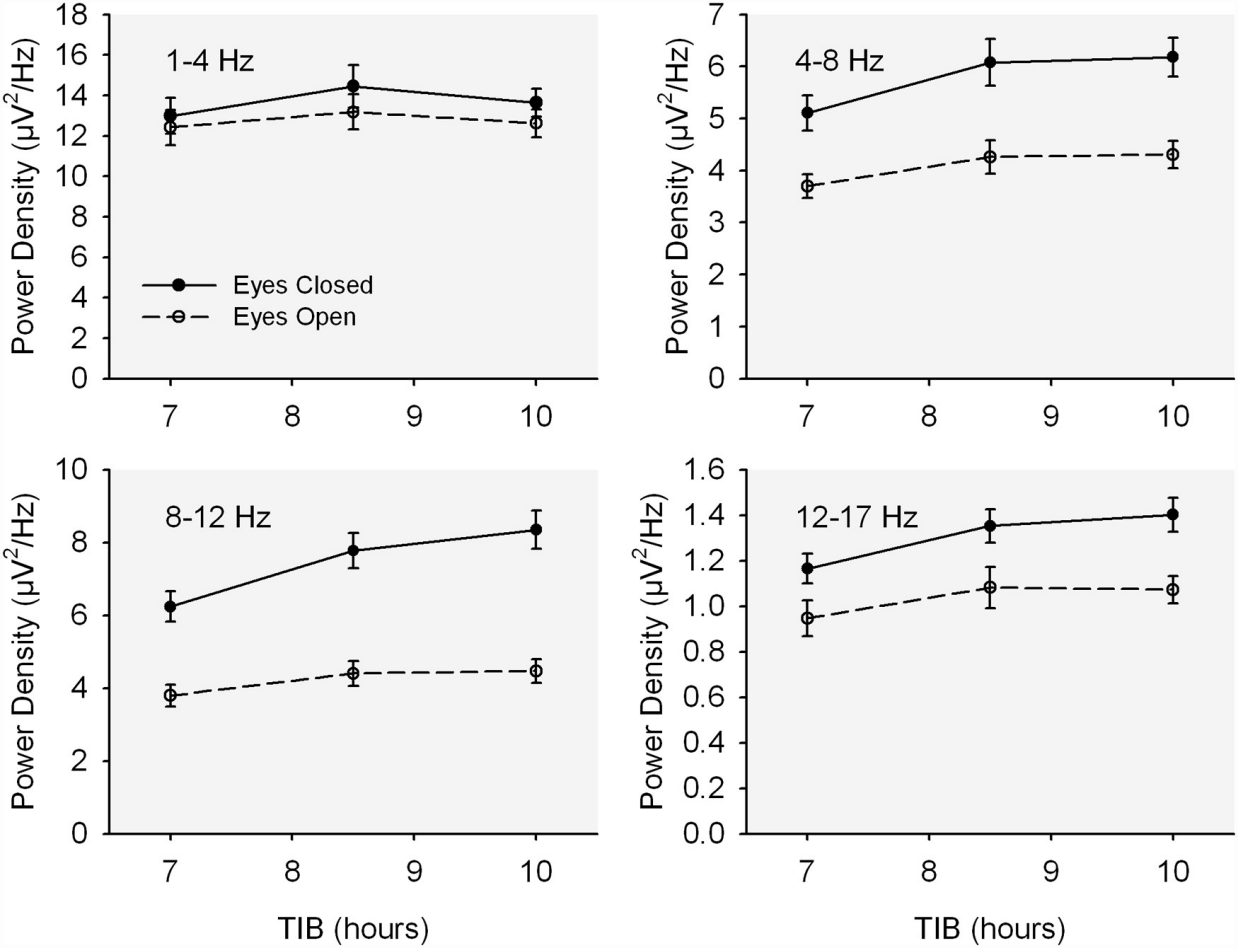
C3 power vs. TIB. The effect of time in bed (TIB) duration on mean (+/- se) C3 waking EEG power density in four frequency bands for both the eyes closed (solid line, filled circles) and eyes open (dashed line, open circles) conditions.

Delta power density in C3, in contrast to delta PD in O1, was not significantly reduced by shorter TIB although PD in theta through low beta in C3 was reduced (Fig 3). For C3, only alpha PD showed a stronger TIB effect with eyes closed. For the other 4 bands TIB and eyes closed effects did not significantly interact.

For both O1 and C3 EEG, there was a highly significant decrease in log power with age in all 5 frequency bands (Table 2).

Replacing TIB with actual sleep durations yielded results similar to those reported for TIB. For example, O1 alpha increased with increasing prior total sleep time (p<0.0001) and eyes closed (p<0.0001), and the sleep duration effects were greater in the eyes closed condition (p<0.0001).

## Discussion

Before addressing the effect of sleep duration on waking EEG activity, we note that our data indicate that eye closure increases EEG power in many waking frequencies in addition to its well-known effect on occipital alpha, and this response occurs in leads anterior to the occipital. These results are consistent with the prior observations of Barry et al [10] and Geller et al [11]. Both groups found that eye closure increases waking EEG power in multiple areas over a wide range of frequencies. Geller et al’s direct brain recordings with subdural electrodes are especially compelling. They found that eye closure increased PD in subgamma frequencies in occipital, parietal, temporal and frontal cortices and both hippocampi, as well as producing focal decreases in high frequency (gamma) power in the occipital lobe. Both Barry et al and Geller et al interpreted the suppression of EEG PD with opening the eyes as evidence of widespread activation of information processing systems, rather than as simple manifestations of changes in global arousal levels. We would add the suggestion that this widespread response to opening the eyes emphasizes the profound importance of visual information for the human brain.

Our data support prior findings [5, 6] that reductions in prior sleep duration are indicated by the Alpha Attenuation Test. Our data further indicate that, at least for adolescents, the AAT can be seen as a specific example of the generalized, but uneven decrease in EEG power with insufficient sleep. These decreases are more pronounced with eyes closed EEG than eyes open, producing the reduced ratio which indicates sleepiness on the AAT.

How might the sleep restriction in this experiment reduce waking EEG power? It is generally accepted that EEG oscillations are produced by synchronous changes in the synaptic potentials of large populations of cortical neurons [12]. The magnitude of these oscillations therefore depends on both the number of neurons oscillating synchronously and the amplitude of the average cortical potential change/neuron. One of the most robust physiological changes during sleep is a 25–40% reduction in whole brain cortical metabolic rate (CMR) during N3 (deep NREM) sleep [3, 4, 13]. In contrast, overall CMR is not below waking levels in REM [13] and is only slightly lower (~5%) in N2 (light NREM) sleep [14]. While depressed cerebral metabolism in N3 must contribute to the restorative function of sleep, this effect could not produce our findings here since sleep restriction did not reduce N3 durations. This finding supports previous findings in adults [15, 16]. Since N3, the major hypometabolic component of human sleep was not reduced, we cannot attribute the decreased waking EEG power to insufficient whole-brain recuperation. However, Braun et al’s studies of regional blood flow show that hypometabolism in certain higher integrative centers (prefrontal and dorsolateral cortex) persists during REM sleep; taken with Madsen et al’s data, it remains possible that loss of N2 and REM sleep (as occurred in our experiment—see Table 1) reduced metabolic recovery in these integrative brain centers. If such regional hypometabolism produced the decrease in waking EEG power, we believe that that this effect more likely resulted from a decrease in the number cortical neurons undergoing synchronous potential change rather than from smaller potential changes per neuron. We recognize the speculative nature of this interpretation and that future understanding of the physiology of sleep recuperation might provide a different explanation of how insufficient sleep duration reduces waking EEG power.

Whatever the underlying mechanism(s), our EEG findings suggest new experiments. Since our subjects were adolescents, it would be important to determine whether, as we expect, the same effect occurs in adults. One would also wish to map these EEG responses with a much more extensive electrode array and with a narrow band examination of the frequency responses. Our observation that reduced sleep duration reduces delta power in O1 but does not produce this effect in C3 indicates that such explorations could be productive. It would also be interesting to determine whether reduced power density with sleep loss is produced by reductions in EEG amplitudes or density in the frequency bands affected. This information could provide clues to the underlying biophysical mechanisms.

On a practical level, changes in power density with varied sleep durations might be used to develop a biological test for sleep sufficiency. If sensitive and specific, such a test could have a range of applications e.g. in research on sleep and cognition, aging, and hypnotic efficacy. It might also help determine whether adolescents are getting adequate sleep at different ages or whether military and public service personnel have obtained the amount of sleep needed to perform critical tasks. Since the EEG changes we report here can be recorded in short sessions and rapidly analyzed, the measurements required for such a test should be technically feasible.

## Supporting information

S1 Fig O2 power spectra. Waking EEG power spectra for O2 with eyes closed and eyes open on the day following 4 consecutive nights of 3 different TIB schedules. Increasing TIB produced an overall increase in power density (F_1,76_ = 27.3, p<0.0001). The TIB effect differed by frequency band (F_44,2.2x105_ = 39.9, p<0.0001). Thick bars above the x-axis indicate a significant (p<0.0001) TIB effect. Thin bars indicate a significant (p<0.01) TIB effect.
(TIF)

S2 Fig O2 power vs. TIB. The effect of time in bed (TIB) duration on mean (+/- se) O2 waking EEG power density in four frequency bands for both the eyes closed (solid line, filled circles) and eyes open (dashed line, open circles) conditions.
(TIF)

S3 Fig C4 power vs. TIB. The effect of time in bed (TIB) duration on mean (+/- se) C4 waking EEG power density in four frequency bands for both the eyes closed (solid line, filled circles) and eyes open (dashed line, open circles) conditions.
(TIF)

S1 Table O2 and C4 statistical analysis. Mixed effect analysis of time in bed (TIB), age, and eyes closed effects on power of waking EEG recorded from O2 and C4. Significance level is bold for positive effects (e.g. increasing power with increasing TIB), italicized for negative effects (e.g. decreasing power with age), and plain text for non-significant (α = 0.01) effects.
(DOCX)

S1 File Results for C4/A1 and O2/A1. (DOCX)
