# Supplementary figures and images for: Shorter sleep durations in adolescents reduce power density in a wide range of waking electroencephalogram frequencies
Source: PLoS One. 2019 Jan 22;14(1):e0210649. doi: 10.1371/journal.pone.0210649 (PMC6342317; doi:10.1371/journal.pone.0210649)

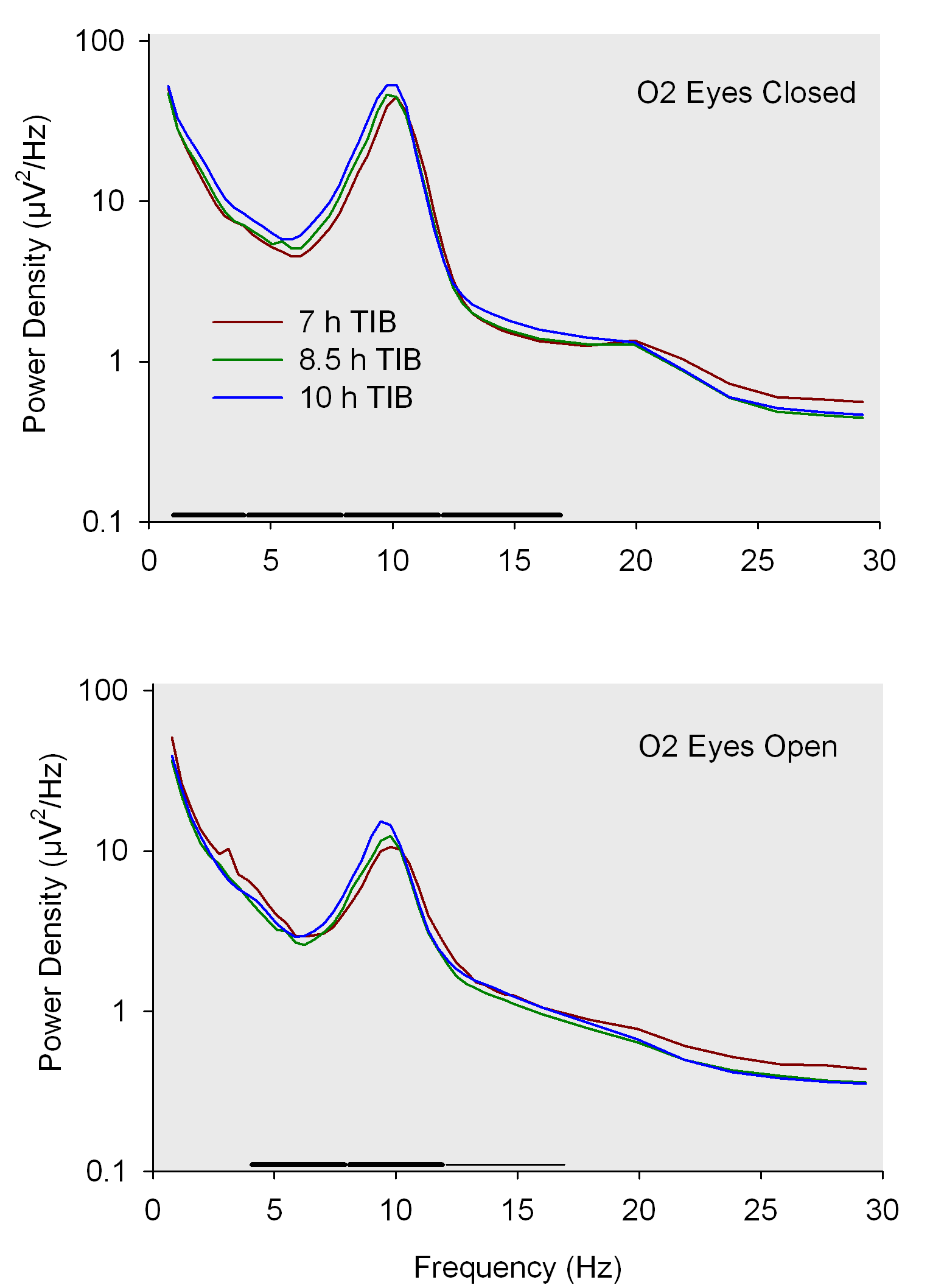

Supplement: S1 Fig — Waking EEG power spectra for O2 with eyes closed and eyes open on the day following 4 consecutive nights of 3 different TIB schedules. Increasing TIB produced an overall increase in power density (F1,76 = 27.3, p<0.0001). The TIB effect differed by frequency band (F44,2.2x105 = 39.9, p<0.0001). Thick bars above the x-axis indicate a significant (p<0.0001) TIB effect. Thin bars indicate a significant (p<0.01) TIB effect. (TIF) [file pone.0210649.s001.tif]

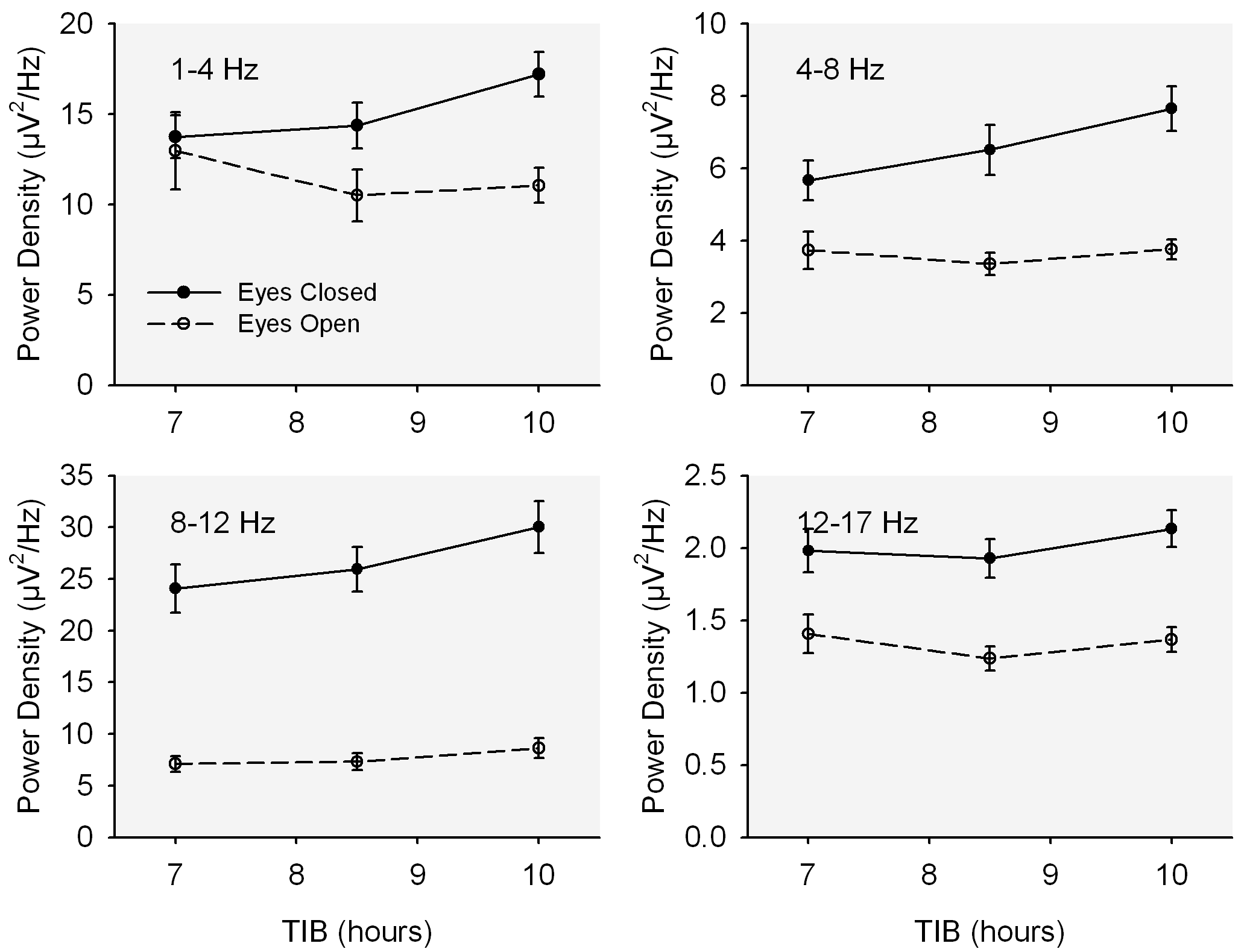

Supplement: S2 Fig — The effect of time in bed (TIB) duration on mean (+/- se) O2 waking EEG power density in four frequency bands for both the eyes closed (solid line, filled circles) and eyes open (dashed line, open circles) conditions. (TIF) [file pone.0210649.s002.tif]

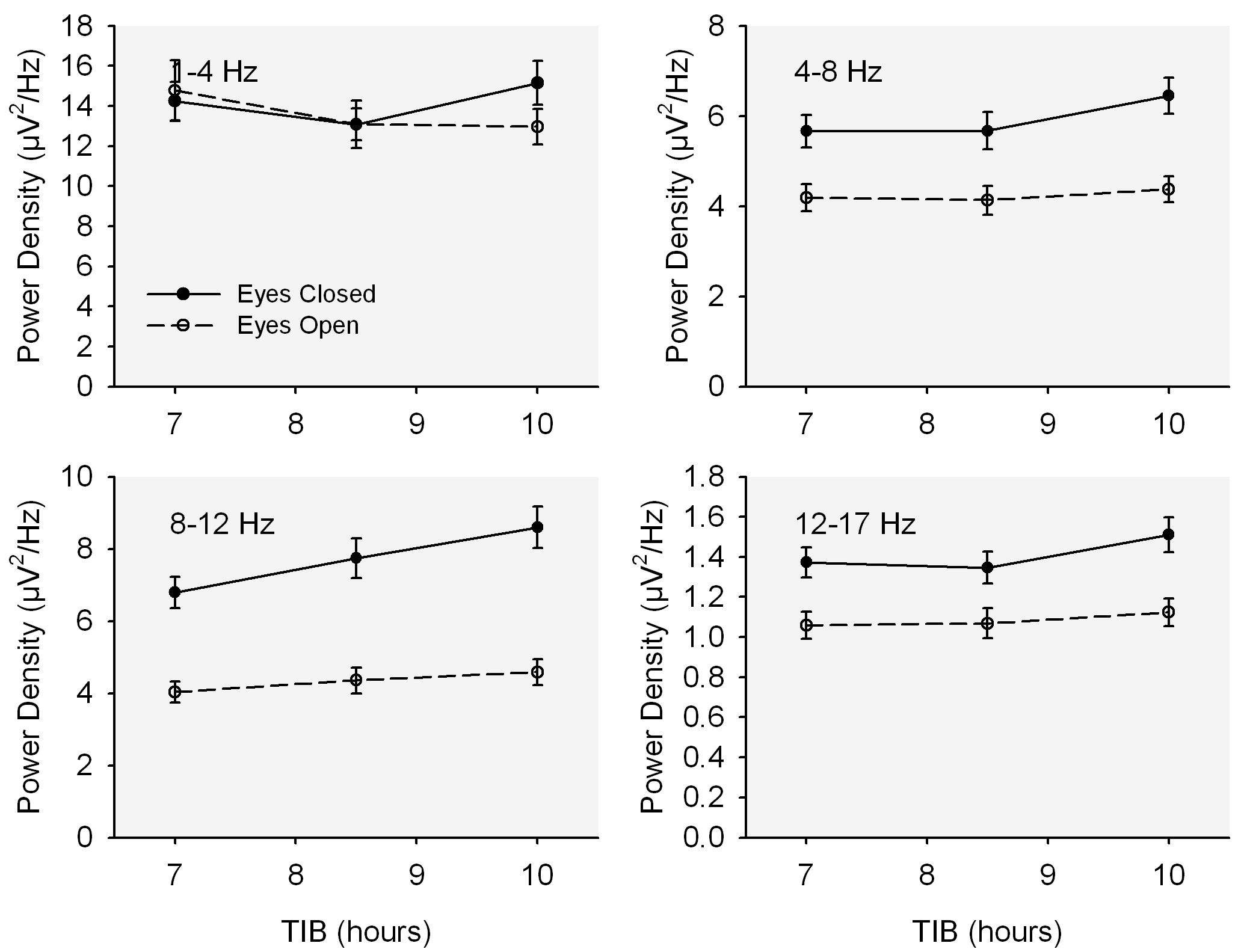

Supplement: S3 Fig — The effect of time in bed (TIB) duration on mean (+/- se) C4 waking EEG power density in four frequency bands for both the eyes closed (solid line, filled circles) and eyes open (dashed line, open circles) conditions. (TIF) [file pone.0210649.s003.tif]
