# Supplementary material for: Shorter sleep durations in adolescents reduce power density in a wide range of waking electroencephalogram frequencies
Source: PLoS One. 2019 Jan 22;14(1):e0210649. doi: 10.1371/journal.pone.0210649 (PMC6342317; doi:10.1371/journal.pone.0210649)
Supplement: S1 Table — Mixed effect analysis of time in bed (TIB), age, and eyes closed effects on power of waking EEG recorded from O2 and C4. Significance level is bold for positive effects (e.g. increasing power with increasing TIB), italicized for negative effects (e.g. decreasing power with age), and plain text for non-significant (α = 0.01) effects. (DOCX) [file pone.0210649.s004.docx]

**S1 Table. O2 and C4 statistical analysis.** Mixed effect analysis of time in bed (TIB), age, and eyes closed effects on power of waking EEG recorded from O2 and C4. Significance level is bold for positive effects (e.g. increasing power with increasing TIB), italicized for negative effects (e.g. decreasing power with age), and plain text for non-significant (α=0.01) effects.

| O2 | | | | |
| --- | --- | --- | --- | --- |
| Band | TIB | Age | Eyes closed | TIB * Eyes |
| Delta 1-4 Hz | **<0.0001** | *<0.0001* | **<0.0001** | **<0.0001** |
| Theta 4-8 Hz | **<0.0001** | *<0.0001* | **<0.0001** | **<0.0001** |
| Alpha 8-12 Hz | **<0.0001** | *<0.0001* | **<0.0001** | **<0.0001** |
| Beta 12-17 Hz | **0.0001** | *<0.0001* | **<0.0001** | 0.097 |
| Beta 17-30 Hz | 0.26 | *<0.0001* | **<0.0001** | 0.096 |
| C4 | | | | |
| Band | TIB | Age | Eyes closed | TIB * Eyes |
| Delta 1-4 Hz | 0.37 | *<0.0001* | **<0.0001** | **0.0021** |
| Theta 4-8 Hz | **0.0057** | *<0.0001* | **<0.0001** | 0.011 |
| Alpha 8-12 Hz | **0.0002** | *<0.0001* | **<0.0001** | **<0.0001** |
| Beta 12-17 Hz | **0.0007** | *<0.0001* | **<0.0001** | 0.13 |
| Beta 17-30 Hz | 0.41 | *<0.0001* | **<0.0001** | 0.049 |
