# Supplementary material for: Shorter sleep durations in adolescents reduce power density in a wide range of waking electroencephalogram frequencies
Source: PLoS One. 2019 Jan 22;14(1):e0210649. doi: 10.1371/journal.pone.0210649 (PMC6342317; doi:10.1371/journal.pone.0210649)
Supplement: S1 File — (DOCX) [file pone.0210649.s005.docx]

**Results for C4/A1 and O2/A1 Waking EEG.**

The main text reports the data for EEG recorded at C3 and O1. As shown in S1 Table and S1 fig, S2 fig, and S3 fig, the results for the right side of the head, C4 and O2, were very similar to those for the left side of the head, C3 and O1. For O2, sleep restriction reduced power density in all bands from delta through beta 1, but did not significantly affect beta 2. For C4, sleep restriction reduced power density in theta, alpha, and beta 1 but not in delta or beta 2. An age related decline in power density was seen across all frequency bands, and the eyes closed related increase in power density was significant for all bands except beta 2 for C4. For many bands, the eyes closed and TIB effects interacted such that the TIB effect was greater for the eyes closed condition.


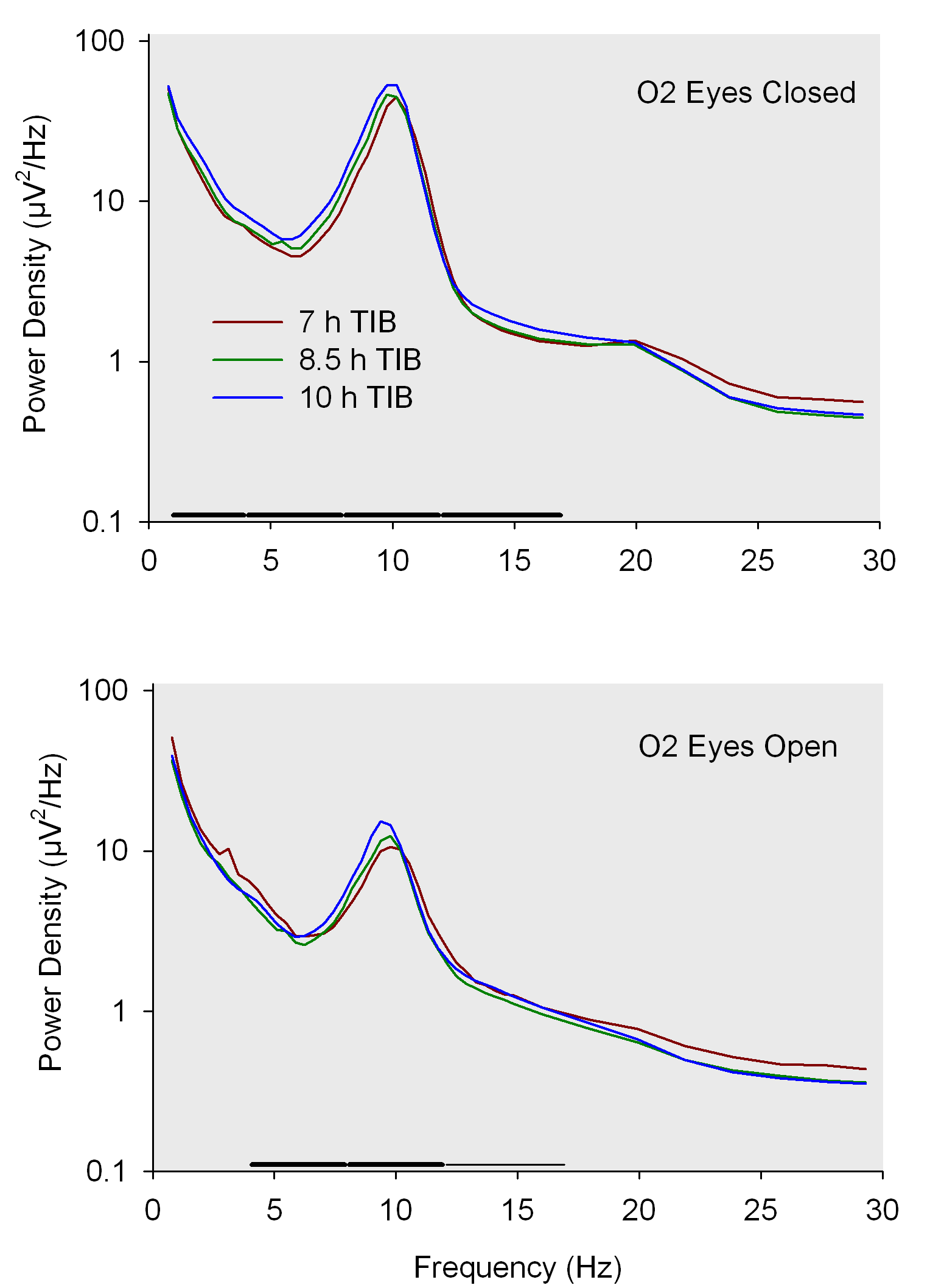


**S1 Fig.** **O2 power spectra.** Waking EEG power spectra for O2 with eyes closed and eyes open on the day following 4 consecutive nights of 3 different TIB schedules. Increasing TIB produced an overall increase in power density (F_1,76_=27.3, p<0.0001). The TIB effect differed by frequency band (F_44,2.2x105_=39.9, p<0.0001). Thick bars above the x-axis indicate a significant (p<0.0001) TIB effect. Thin bars indicate a significant (p<0.01) TIB effect.


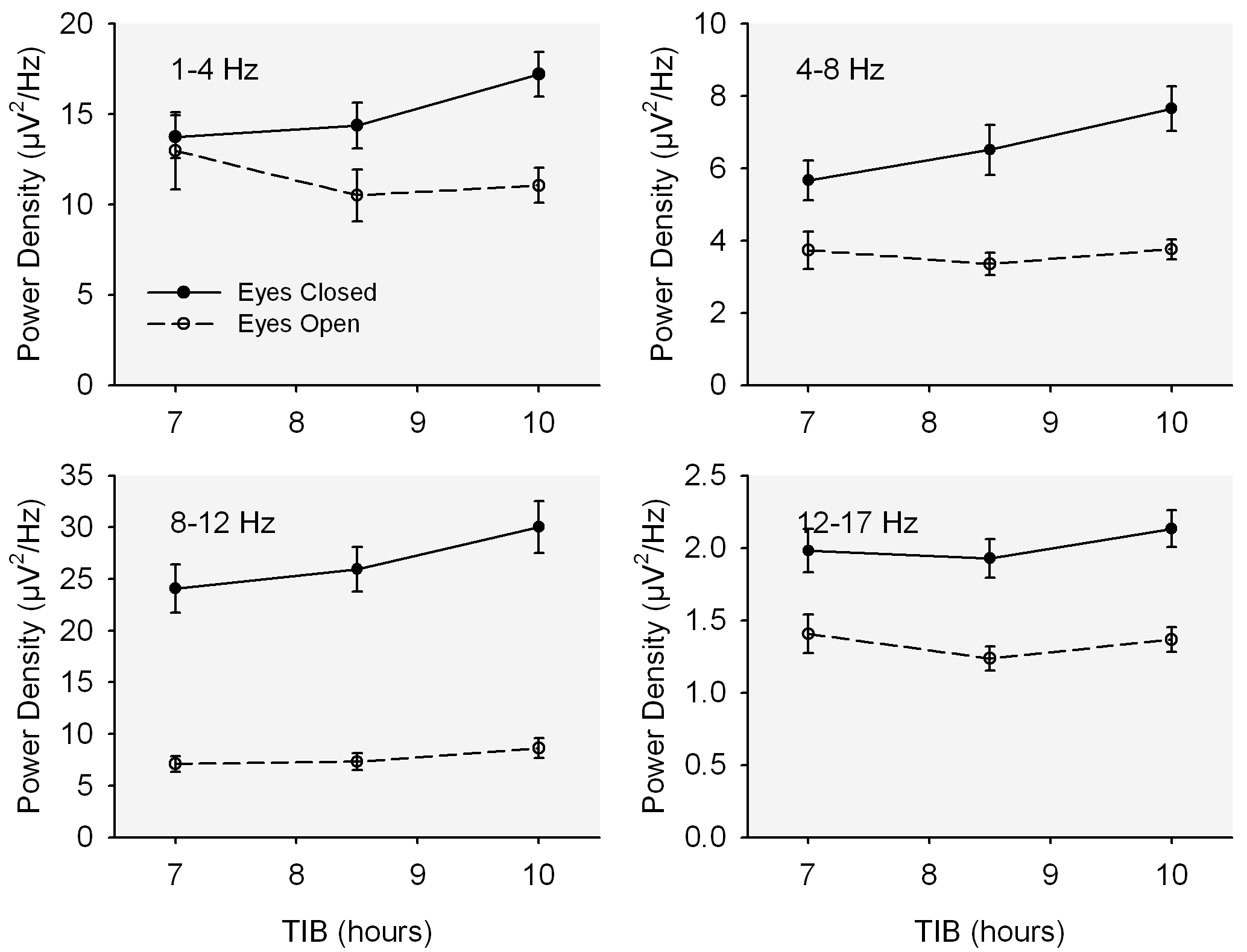


**S2 Fig.** **O2 power vs. TIB.** The effect of time in bed (TIB) duration on mean (+/- se) O2 waking EEG power density in four frequency bands for both the eyes closed (solid line, filled circles) and eyes open (dashed line, open circles) conditions.


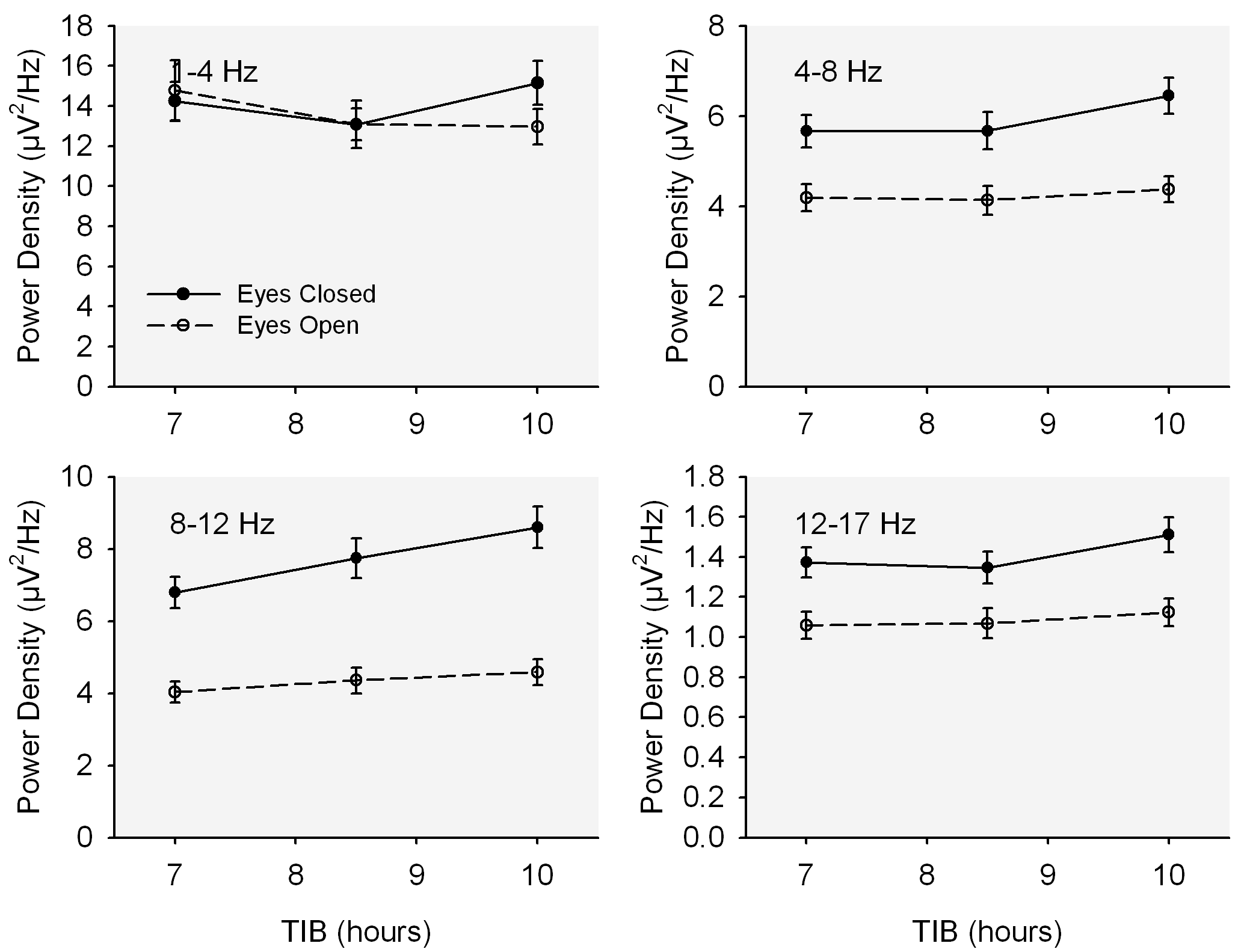


**S3 Fig.** **C4 power vs. TIB** The effect of time in bed (TIB) duration on mean (+/- se) C4 waking EEG power density in four frequency bands for both the eyes closed (solid line, filled circles) and eyes open (dashed line, open circles) conditions.

**S1 Table**. **O2 and C4 statistical analysis**  Mixed effect analysis of time in bed (TIB), age, and eyes closed effects on power of waking EEG recorded from O2 and C4. Significance level is bold for positive effects (e.g. increasing power with increasing TIB), italicized for negative effects (e.g. decreasing power with age), and plain text for non-significant (α=0.01) effects.

| O2 | | | | |
| --- | --- | --- | --- | --- |
| Band | TIB | Age | Eyes closed | TIB * Eyes |
| Delta 1-4 Hz | **<0.0001** | *<0.0001* | **<0.0001** | **<0.0001** |
| Theta 4-8 Hz | **<0.0001** | *<0.0001* | **<0.0001** | **<0.0001** |
| Alpha 8-12 Hz | **<0.0001** | *<0.0001* | **<0.0001** | **<0.0001** |
| Beta 12-17 Hz | **0.0001** | *<0.0001* | **<0.0001** | 0.097 |
| Beta 17-30 Hz | 0.26 | *<0.0001* | **<0.0001** | 0.096 |
| C4 | | | | |
| Band | TIB | Age | Eyes closed | TIB * Eyes |
| Delta 1-4 Hz | 0.37 | *<0.0001* | **<0.0001** | **0.0021** |
| Theta 4-8 Hz | **0.0057** | *<0.0001* | **<0.0001** | 0.011 |
| Alpha 8-12 Hz | **0.0002** | *<0.0001* | **<0.0001** | **<0.0001** |
| Beta 12-17 Hz | **0.0007** | *<0.0001* | **<0.0001** | 0.13 |
| Beta 17-30 Hz | 0.41 | *<0.0001* | **<0.0001** | 0.049 |
